# Recurring genomic breaks in independent lineages support genomic fragility

**DOI:** 10.1186/1471-2148-6-90

**Published:** 2006-11-07

**Authors:** Hanno Hinsch, Sridhar Hannenhalli

**Affiliations:** 1 Penn Center for Bioinformatics, University of Pennsylvania, Philadelphia, USA; 2 Department of Genetics, University of Pennsylvania, Philadelphia, USA

## Abstract

**Background:**

Recent findings indicate that evolutionary breaks in the genome are not randomly distributed, and that certain regions, so-called *fragile regions*, are predisposed to breakages. Previous approaches to the study of genomic fragility have examined the distribution of breaks, as well as the coincidence of breaks with segmental duplications and repeats, within a single species. In contrast, we investigate whether this regional fragility is an inherent genomic characteristic and is thus conserved over multiple independent lineages.

**Results:**

We do this by quantifying the extent to which certain genomic regions are disrupted repeatedly in independent lineages. Our investigation, based on Human, Chimp, Mouse, Rat, Dog and Chicken, suggests that the propensity of a chromosomal region to break is significantly correlated among independent lineages, even when covariates are considered. Furthermore, the fragile regions are enriched for segmental duplications.

**Conclusion:**

Based on a novel methodology, our work provides additional support for the existence of fragile regions.

## Background

Genome evolution is carried out via molecular events varying in size and scope from single base substitutions to large scale chromosomal shuffling. This global view of evolution, based on large and relatively infrequent events provides an alternative means to infer evolution [1-5]. Each rearrangement event involves 'breaking' and rejoining of the genomic DNA. These events can be inferred by an analysis of genomes sharing a common ancestor: given two permutations representing the common marker (*e.g *.. gene) orders in two genomes, if two adjacent markers in one genome are not adjacent in the other genome, the "region" between the two markers is called a *breakpoint*. The exact location where the break occurs in this region is usually unknown. A maximal genomic segment devoid of breakpoints is called a *synteny block*, or *block*. A question of fundamental interest is – what are the forces behind the choice of genomic breakpoints? Specifically, are breakpoints chosen randomly or are there genomic locations more prone to breakage than others, and why?

Despite the initial report by Nadeau and Taylor which supported a random (uniform and independent) breakage model [6] , a number of studies since then have argued to the contrary [7-9]. For instance, Pevzner and Tesler showed that the synteny-block-size distribution estimated by random breakage model does not hold if one considers small blocks revealed by recent high-resolution human-mouse comparative data [7]. They estimated that each breakpoint region was broken 1.9 times on an average and the additional breaks were distributed non-uniformly among the breakpoint regions– suggesting hotspots. They also suggested that sequence 'churning' in the breakpoint regions, as observed in examinations of sequence alignments, is additional evidence of fragility. However, it was later argued that both the additional breakpoints as well as the sequence churning can be explained, at least partly, by inadvertent exclusion of small blocks [10] and artifacts introduced during the reconstruction of blocks [11]. An association between evolutionary breaks and segmental duplications has been previously reported [8,12] suggesting that specific regions within the genome have been predisposed to small duplications as well as large evolutionary rearrangements. This was also found to be true in a mouse-rat comparison relative to rodent specific segmental duplications [9]. Hotspots have also been observed in other independent contexts, *e.g*., HIV integration sites [13] , chromosomal breakages in various cancers [14,15] and other diseases [16].

Existing arguments supporting predisposition of certain regions for breakage are based on the observed co-incidence or clustering of independent breakage events (*e.g*., large rearrangements, segmental duplications, or breaks in tumor cells) on a genome. These analyses, however, cannot rule out random breakage followed by negative selection. Here we investigate whether this predisposition is conserved over long evolutionary periods spanning multiple lineages, and further strengthen the previous arguments. We use a novel approach to assert independence among the evolutionary breaks, while minimizing the role of negative selection as an underlying cause. Specifically, we search for genomic regions that have undergone breakage in multiple lineages (hence independently), as an overabundance of such regions would support the existence of fragile regions. In this paper, we use the term '*fragile*' to imply a vulnerability of the chromosomal region for breakage in an evolutionary time scale, and not necessarily something that leads to pathological condition in an individual, as is the case in medical genetics.

A recent paper considered a breakpoint region in human as *reused *if the breakpoint region overlaps with one or more but not all species from different orders [5]. Additionally, depending on the level of overlap, it classifies breakpoint regions into *lineage-specific, order-specific, superordinal *and *reused*. We strengthen this by imposing an additional restriction that the breakpoint regions be flanked by homologous blocks. Also, we specifically examine the correlation among the species-specific and order-specific breaks, *i.e*., the ones which can be unambiguously mapped to a specific species or order (such as rodent or primate) branches assuming parsimony in evolutionary breakages. Our analysis is based on a genomic alignment of Human, Chimpanzee, Mouse, Rat, Dog and Chicken.

**Figure 1:**
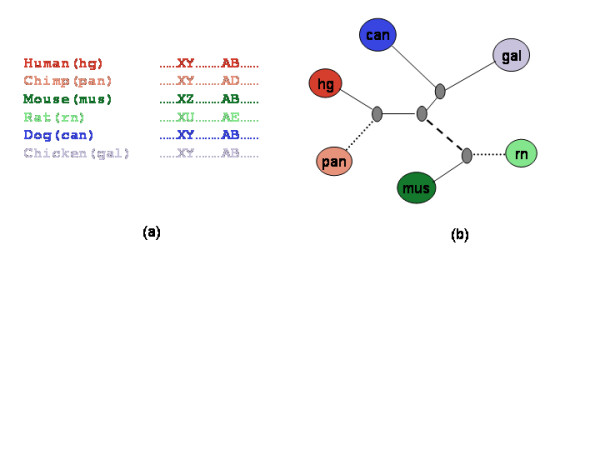
The scheme to detect independent breakage of a region. (a) The region between human markers X and Y is conserved in chimpanzee, dog and chicken but is disrupted in mouse and rat. (b) Under the assumption of parsimony, all species breaks between X and Y can be explained by a single break in the rodent lineage (denoted by the dashed line). The region between human markers A and B in (a) is conserved in mouse, dog and chicken but disrupted in chimpanzee and rat. This can only be explained by two 'independent' breaks in chimpanzee and rat lineages (denoted by dotted lines).

Our main methodological contribution is the way in which we assess the fragility. Consider a set of markers common to the 6 species in Figure 1. Given two species, successive markers are either consecutive in the same orientation in both the species (adjacent), or not (a break). If the markers in the two species are adjacent, and yet if in a third species the markers are not adjacent, then we assert, using a parsimony argument, that the break occurred in the lineage between that third species and its ancestor on the unique evolutionary path between the two species. If we now examine a fourth species and find that it too shows a break, yet does not share a common branch with the other broken genome then we assert that we have identified two independent breaks of the same ancestral sequence (see Figure 1). (A similar idea was used to localize a break within the mouse-specific lineage or a rodent lineage (common to mouse and rat) in three species [8].) Given a genome wide set of markers homologous in multiple species (but in different order and orientation), we study the overall prevalence of fragile regions by measuring the frequency of breaks of the same region in multiple independent lineages and by comparing this frequency against a null model which accounts for possible covariates such as region length and functional class (by removing exons from our analysis). Our results, based on an analysis using two different models and a large parameter space, suggest that the propensity of a chromosomal region to break is significantly correlated among independent lineages. Besides making a methodological contribution, our result provides further evidence for fragile regions, indicates that fragility is at least in part an inherent attribute of a chromosomal region, and further indicates this attribute is conserved across long evolutionary periods spanning multiple lineages. Consistent with previous studies, the fragile regions, detected using our alternative approach, are enriched for segmental duplications, although based on a small sample.

## Results

### Terminology

A *marker *is a stretch of genomic sequence that can be uniquely mapped to a location on a genome. Markers common to multiple species are *homologous *(assumed to be derived from common ancestral sequence). Consider a set M of *n *markers – (*m*_1_*, m*_2_,...,*m_n_*), common to a set of genomes under consideration. Each genome *X *can be represented by a permutation on *M *as G_x _= *(x_1_x_2_*...*x_n_)*. If markers x_i _and x_i+1 _are not adjacent in G_y_, then the region between x_i _and x_i+1 _is called a *breakpoint region *or *breakpoint *in genome *X *with respect to genome *Y*. In all of our discussions we will assume the markers to be on positive strand with implicit understanding that marker order (a b c) is identical to order (-c -b -a). This distinction, although important, is not relevant to the discussion and simplifies the exposition. A (maximal) *syntenic block *of size *k *in genome *X *with respect to genome *Y *is (maximal) sequence of markers (x_i _.... x_i+k_) not containing any breakpoint.

### Identifying independent breakage events

Consider four genomes- Human (*hg*), Chimpanzee (*pan*), Rat (*rn*), Mouse (*mus*). Suppose we have two markers, A and B, and that these two markers are adjacent in human and mouse, but not adjacent in chimpanzee and rat. Therefore two separate breakage events can be unambiguously placed on the chimpanzee and the rat branches. We consider all such combinations of species in which an adjacency in 2 genomes (called the '*fixed' *species; human and mouse in the above example), allows an unambiguous placement of independent breakage events in the branches incident to the two other species (called the '*variable' *species; chimpanzee and rat in the above example). Not all species combinations allow such unambiguous placement of independent breakage events. For example if two markers remain adjacent in human and chimpanzee, breaks between these markers in other species may not be unambiguously placed on a species branch. For the sake of completeness we also consider the species combinations in which the breaks can be unambiguously placed onto an *order *(for example, rodent or primate), if not a *species*. Our conclusions do not change if we exclude these latter combinations. In Figure 1 human and mouse are '*fixed*' species and chimpanzee and rat are '*variable*' species. Denote this combination by *pan,rn|hg,mus*. For all pairs of markers that are adjacent in human and mouse, we count the number of combinations in which the two markers are not adjacent in both, chimpanzee and rat. Denote this count by *β(pan,rn|hg,mus)*. For a given combination of fixed and variable species we compute *β *and then estimate the probability *β^P ^*of achieving a count of *β *based on null hypothesis as described in the methods section.

### Models and parameters

In order to minimize the effect of noise in the data caused by alignment errors, and to test the robustness of the conclusions, we used two different models to decide which regions to consider for the calculation of *β*, and also to decide if a region is considered a break. One model considers only breaks between two consecutive markers (Fragility of specific BreakPoints or *FBP*), while the other model considers breaks in a larger region flanked by anchoring markers (Fragility of Breakpoint Regions or *FBR*). Next we describe these two models.

### Fragility of specific breakpoints (FBP)

**Figure 2:**
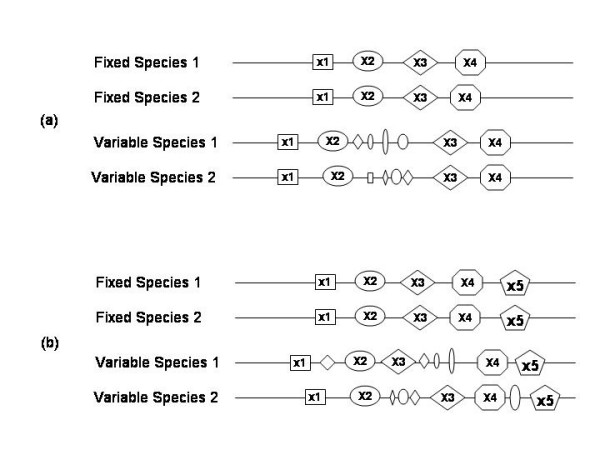
Illustration of 'joint breaks' in the FBP and FBR schemes. Distinct markers are identified by their shape and size. (a) FBP: Markers (*x*_1 _*x*_2 _*x*_3 _*x*_4_) occur in the specified order and consecutively in both of the 'fixed' species. In both 'variable' species, the flanks (*x*_1 _*x*_2_) and (*x*_3 _*x*_4_) are consecutive but the region between markers x_2 _and x_3 _is disrupted (contains additional markers). (b) FBR: Markers (*x*_1 _*x*_2 _*x*_3 _*x*_4 _*x*_5_) occur in the specified order and consecutively in both of the 'fixed' species. In the variable species 1 the flanks (*x*_2 _*x*_3_) and (*x*_4 _*x*_5_) remain unbroken, while in variable species 2 the flanks (*x*_1 _*x*_2_) and (*x*_3 _*x*_4_) remain unbroken, but in both variable species the entire block of 5 markers is disrupted. This situation will be identified as a joint break according to FBR but not according to FBP. FBP is a special case of FBR.

Here we analyze the region between a pair of adjacent markers. We search for say, 4 markers (*x_1 _x_2 _x_3 _x_4_*) that occur in the specified order and consecutively in both of the 'fixed' species. For each of the variable species *V*, we define the breakpoint region between markers x_2 _and x_3 _to be 'broken' if both (*x_1 _x_2_*) and (*x_3 _x_4_*) are consecutive in V but markers x_2 _and x_3 _are not consecutive in *V *(see Figure 2). Note the requirement for consecutive markers on either side of the breakpoint. This is meant to make the analysis more robust against noise. The parameter *'minimum flank' *is the number of required consecutive markers on each side. We have used two values – 2 or 3.

### Fragility of regions (FBR)

The above model – FBP – misses the cases in which breaks in the variable species are not bounded by a single pair of markers, but are nevertheless within a short distance of one another (Figure 2). Such a clustering of breaks would also suggest fragility of the region. From this perspective we search in the maximal blocks in the pair of fixed species – (*x*_1_*x*_2 _...*x_i_x_i+1 _*...*x_k_*). For each of the variable species *V*, we define this block to be 'broken' if there exist two regions within the block, say (*x_i_x_i+1 _*...*x_i+F-1_*) and (*x_j_x_j+1 _*...*x_j+F-1_*),*j>i+F-1*, that are consecutive in *V *but the region (*x_i_x_i+1 _*...*x_j+F-1_*) is not consecutive. This implies a break between markers *x_i+F-1 _*and *x_j_*. FBP is a special case of FBR (Figure 2). The parameter '*F*' is the *minimum flank *as defined for FBP. Besides allowing for a variable breakpoint within the block, note that in FBR we search in maximal blocks rather than blocks of fixed size. Because of this maximality constraint, the number of breakpoint regions is considerably smaller relative to FBP. As in FBP, a value of 2 or 3 is used for the *minimum flank *parameter.

### Parameters

Here we briefly discuss the parameters used to investigate the joint breakages, mainly to ensure the robustness of our conclusions. The following parameters apply to both – FBP and FBR.

1. *Marker length:*Initial filter used to select the common set of marker in six species. We have used a threshold of either 100 bps (when chicken is included) or 500 bps when only mammalian species are included.

2. *Minimum flank:*Minimum number of flanking syntenic markers on either side of the considered break. We have used a value of 2 and 3.

3. *Length differential:*Maximum allowed ratio between the lengths of the homologous blocks in the two fixed species. We have used a value of 0.5 for this parameter. In other words we only consider the blocks whose lengths (in bps) in the two fixed species are within 50% of each other. Large size differences suggest the ancestral sequence has been subject to strongly differing forces in the two lineages, thereby resulting in large unmapped regions, and possibly confounding any inferences based on the assumption of similarity. The *length differential *parameter attempts to minimize such effects.

4. *Rank differential:*Consider two consecutive markers in the fixed species. Let these correspond to *m_i _*and *m_i+k _*in a variable species. Clearly *k *> 1. When *k *= 2, this would nominally be considered a break. However, we have noticed a series of markers (*m_i _m_i+2 _m_i+4_*...) in one genomic location and the missing markers (*m_i+1_m_i+3 _m_i+5_*...) in another location. This apparent interleaving could be due to duplication of a region followed by intermittent degradation of different parts of the two copies. In this case the region between *m_i _*and *m_i+2 _*does not correspond to a break. To avoid wrongly considering sequence degradation in paralogous copies as breaks, we require *k *> 3 for the pair of markers to be considered a break. This minimum required value for *k *is called the '*Rank differential*'. In all of our analyses we have used 2 different values for *Minimum flank *with *Rank differential *set to 3.

### The markers common to multiple species

The marker set was obtained from the six species projection of the 8-way Multiz alignment [17] from the UCSC genome resource. The specific builds of the six species were as follows: Human (hg17), Chimpanzee (panTro1), Mouse (mm5), Rat (rn3), Dog (canFam1), and Chicken (galGal2). Although the common interspersed repeats were already removed prior to the alignment, low copy repeats or segmental duplication may still cause mis-alignments. To minimize this effect we excluded markers that overlap human segmental duplications (see methods); however, this did not change our results. Additionally, in order to account for the potential negative selection against breaks in genes, we excluded from our analysis any block that overlapped gene boundaries (exons and introns). We ran a series of analyses using markers common to different sets of species – (i) 6 species (hg, pan, mus, rn, canis, gal), (ii) 5 species excluding chimpanzee, and (iii) 4 species excluding chimpanzee and chicken. Table 1 shows basic statistics on the marker lengths and inter-marker distances in different species.

**Table 1:** Marker and block statistics

		**Marker Length (bp)**						**Inter-marker distance (kb)**					
		
		*Hg*	*Pan*	*Can*	*Mus*	*Rn*	*Gal*	*Hg*	*Pan*	*Can*	*Mus*	*Rn*	*Gal*
**M6**	*Mean*	354	354	355	347	346	355	19	19	15	16.5	17.8	6
	*Std*	290	290	292	287	287	292	156	119	83	94	108	30
	*Median*	263	263	263	257	256	263	2.7	2.7	2.4	2	2.3	1.3
	*Max*	7789	7786	7794	7754	7753	7888	26399	12395	10076	10978	12199	4307

**M5**	*Mean*	354	na	355	348	346	355	17	na	14	15	16	5.5
	*Std*	293	na	295	290	290	295	136	na	73	81	95	26
	*Median*	263	na	263	257	256	263	2	na	2.4	2	2.3	1.3
	*Max*	11849	na	11854	11814	11813	11957	26399	na	10076	6343	9852	2586

**M4**	*Mean*	990	na	981	937	932	na	4.7	na	3.5	3.9	4.3	na
	*Std*	569	na	569	548	546	na	55	na	11	19	26	na
	*Median*	815	na	809	772	768	na	1.3	na	1	1.3	1.3	na
	*Max*	17901	na	18273	17949	18129	na	20538	na	2176	2868	6050	na

Marker and block statistics for various studies. M6: Markers common to 6 species (hg, Pan, Mus. Rn, Cannis, Gal). M5: Markers common to 5 species excluding Pan. M4: Markers common to 4 species excluding Pan and Gal.

### Estimating the significance of correlated breaks in independent lineages

Two independent events occurring with probabilities *p *and *q *are expected to co-occur with probability *p.q*. A deviation from this expectation indicates dependence. In our application, the events correspond to breakage at a specific branch. The question we are interested in is whether breakage of a region at a specific branch (event 1) co-occurs with a breakage of the same region in another branch (event 2) more frequently than one would expect by chance alone. With reference to the markers *A *and *B *in Figure 1, let the *fixed *species be *hg *and *mus *and the *variable *species be *pan *and *rn*. In this case the status of *A *and *B *in *gal *and dog is irrelevant. Given the parameters, let *s *be total number of blocks (in either FBP or FBR) syntenic in the fixed species (for FBP, these blocks may be overlapping). In each of the *s *blocks, we then identify whether a break occurred in the variable species using the criteria defined above. Note that many blocks might be completely disturbed in the variable species, but may not be considered a break unless the break can be confidently localized in the variable species by virtue of syntenic flanking markers. Each of the *s *blocks thus corresponds to a binary tuple – (b_1 _b_2_) – where b_1 _(respectively b_2_) is 1 if the block was broken in variable species 1 (respectively 2) and 0 otherwise. A value of 1 corresponds to the occurrence of the event. The number *β *of tuples of the form (1,1) represents the number of joint breaks, *i.e*., co-occurrence of the two events. We compare *β *against a carefully designed shuffled data set. Our shuffling scheme is described in the methods section. Below we present the intuition behind it.

We are given *s *binary tuples, *i.e*., matched events, *β *of which are (1,1). If the two events are un-correlated, permuting one of the events relative to the other should not significantly affect *β*. Thus generating random permutations and calculating the fraction of permutations with equal or greater value than *β *gives us a significance value of *β*. This is a standard permutation test for significance. This test is appropriate if the *a priori *probability of an event is uniform over all events. In our application, however, this probability increases with the length of the genomic region. Thus, we need to modify our permutation scheme to reflect this non-uniform event probability. We do this as follows: Within each species we sort the blocks by the length of their breakpoint regions. We then generate a new set of tuples, creating each new tuple by pairing the first element of the original tuple with a randomly chosen block from the other species whose breakpoint length is within certain threshold of the length of the original block. We use 200 bp or 20% of the original length as the threshold, whichever is greater. We have also implemented an additional strategy where we by pairing the first element of the original tuple with a block whose length is strictly greater than that for the original (see Additional file 1). We generate 1000 random sets of matched blocks in the two variable species. The number of times, among 1000 shuffles, that the calculated number of joint breaks exceeds *β *provides a measure of significance *β^p^*. For instance if this number exceeds *β *only once among 1000 shuffle then *β^p ^*= 0.001. We consider a correlated break frequency *significant *if *β^p ^*≤ 0.05.

### Correlated breaks using markers common to human, chimpanzee, dog, mouse, rat, and chicken

First we estimated the significance of correlated breaks for various combinations of fixed and variable species among the six species and the 4 parameter combinations described above. For this analysis, we have used a minimum marker length threshold of 100 bps. Table 1 shows the basic statistics of marker lengths and inter-marker distances in different species.

For various parameter and lineage combinations, Table S1a,b (see Additional file 1) show the statistics on correlated breaks, for FBP, and FBR, for a total of 88 experiments with each model (2 parameters and 44 species combinations). At the stringent parameter combinations, often there are very few breaks in the variable species to reliably estimate significance of joint breaks. We require a minimum of 10 breaks in each of the variable species. In case of FBP, of the 63 combinations that meet this criterion, 30 (48%) have significantly correlated breaks (*i.e*., with p-value ≤ 0.05), and in FBR, of the qualifying 46 combinations 30% are significant. Note that at a significance level of 0.05, we expect only 5% of the combinations to show significance. Upon inspection of the results, we noticed two trends.

**Figure 3:**
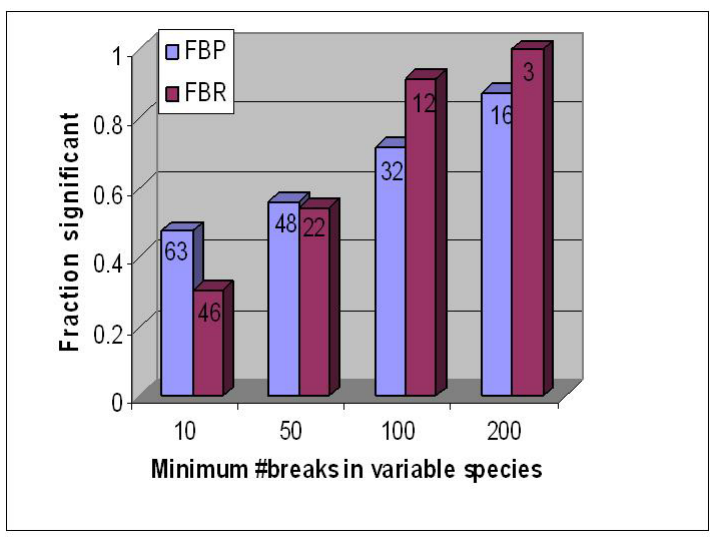
A plot of the fraction of species combinations showing significance of correlated breaks (Y-axis) against the threshold for number of breaks in the variable species (X-axis). This is based on the 6 species analysis. The same trend follows in other analyses as well. As we consider combinations with more breaks in the variable species, the fraction that show significance grows steadily. (a) FBP, (b) FBR. The numeric label on each bar indicates the number of combinations that are above the threshold.

First, the fraction of significant combinations increases with the number of breaks in the variable species. To highlight this, we applied an increasing threshold for the minimum number of breaks in a variable species and for each such threshold we determined the fraction of significant combinations. Figure 3 shows this. For instance, when we require that the variable species have at least 200 breaks each, the fraction of significant combinations in FBP and FBR are 87% and 91% respectively.

The second trend we noticed is that the combinations involving chimpanzee are frequently less significant. For FBP, for the combinations not involving chimpanzee, 59% of the 34 qualifying combinations are significant, while among the combinations involving chimpanzee only 34% of the 29 qualifying combinations are significant. As our definition of a break under the FBR model is stricter, the chimpanzee versus non-chimpanzee skew in the fraction of significant combinations in FBR is not nearly as drastic as in FBP. Upon manual inspection of the broken regions in chimpanzee on the UCSC browser, we found that every broken region in chimpanzee had significant gaps, and was composed of many contigs. This observation is consistent with other assessments of the relatively poor assembly of the chimpanzee genome [18] (N50 contig length is only 15.7 Kb). An additional problem is that the chimpanzee genome was not assembled de novo but based on the human assembly, and thus some real chimpanzee rearrangements may have been lost. Because of these problems with the chimpanzee assembly we repeated our analysis after excluding chimpanzee.

### Correlated breaks using markers common to human, mouse, rat, dog, and chicken

For this analysis we used a minimum marker length threshold of 100 bps. Table 1 shows the basic statistics of marker lengths and inter-marker distances in different species. For various parameter and species combinations, Table S2a,b (see Additional file 1) show the statistics on correlated breaks, for both FBP and FBR. There are a total of 40 experiments performed for each model (2 parameters and 20 species combinations). As before we require a minimum of 10 breaks in each of the variable species. Using FBP, of the 34 qualifying combinations 47% are significant, and using FBR, of the 24 qualifying combinations 33% are significant. Table 2 shows the combinations for *Minimum flank *= 2 for FBP, for which 55% of the 20 qualifying combinations are significant.

**Table 2:** Analysis of markers common to 5 species

**Fixed species**	**# blocks**	**Variable species 1 (# breaks)**	**Variable species 2 (# breaks)**	**#joint breaks **(***β***	**Avg #joint breaks in shuffles**	**p-value of correlated break **(***β^p^*)**
hg,mus	126798	canis(127)	rn(421)	3	2.4	0.441
hg,rn	122981	canis(111)	mus(69)	0	0.3	1
canis,mus	121649	hg(81)	rn(416)	0	1.8	1
canis,rn	117204	hg(84)	mus(69)	1	0.3	0.286
hg,mus	126798	gal(1224)	rn(421)	33	19.1	0.002
hg,rn	122981	gal(1152)	mus(69)	6	2.6	0.036
gal,mus	47121	hg(17)	rn(60)	0	0.1	1
gal,rn	46049	hg(19)	mus(19)	1	0	0.01
canis,mus	121649	gal(1253)	rn(416)	35	19.8	0.001
canis,rn	117204	gal(1187)	mus(69)	8	3	0.011
gal,mus	47121	canis(20)	rn(60)	1	0.1	0.126
gal,rn	46049	canis(19)	mus(19)	1	0	0.047
canis,hg	123305	gal(1278)	rn(582)	67	30.5	0
canis,rn	117204	gal(1187)	hg(84)	9	4.4	0.05
gal,hg	38367	canis(17)	rn(55)	0	0	1
gal,rn	46049	canis(19)	hg(19)	3	0	0
canis,hg	123305	gal(1278)	mus(229)	37	12.3	0
canis,mus	121649	gal(1253)	hg(81)	8	4.4	0.081
gal,hg	38367	canis(17)	mus(30)	0	0.1	1
gal,mus	47121	canis(20)	hg(17)	1	0	0.021

For Minimum flank = 2 and Rank differential = 3, the table shows the number of breaks in the variable species, the number of joint breaks, the average number of joint breaks in 1000 random shuffles and the p-value of the joint breaks. This is based on greater than 100 bps markers common to Human, Mouse, Rat, Dog and Chicken. This is based on FBP. In all 20 combinations both variable species have at least 10 breaks and 55% exhibit significant joint breaks.

The fraction of significant combinations in FBR is much lower than that in FBP. We have argued that this is most likely due to fewer breaks in variable species in the case of FBR. The trend illustrated in Figure 3 for the 6 species hold precisely for the 5 species as well. Although the chicken genome sequence assembly is of relatively high quality, because of a greater evolutionary distance of chicken from the other species in the set, there are greater number of breaks in chicken relative to other species and consequently, a larger fraction of combinations involving chicken as a variable species are significant.

### Correlated breaks using markers common to human, mouse, rat, and dog

Because of a large number of markers common to only 4 species, we had to apply a minimum length threshold of 500 bps in this analysis. In contrast to the 5 species case, both for FBP and FBR, the number of breaks in the variable species is too small relative to the number of blocks to achieve a significance in correlated breaks (data not shown). In FBP, only one of the 8 combinations is significant. However, if we reduce the *Rank Differential *threshold to 2 (see above), thereby increasing the number of breaks considered, as shown in Table 3, four of the 8 combinations are significant.

**Table 3:** Analysis of markers common to 4 species

**Minimum Flank**	**Fixed species**	**# blocks**	**Variable species 1 (# breaks)**	**Variable species 2 (# breaks)**	**#joint breaks (*β***	**Avg #joint breaks in shuffles**	**p-value of correlated break (*β^p^*)**
2	hg,mus	403240	canis(741)	rn(1067)	17	10.4	0.025
2	hg,rn	394250	canis(709)	mus(451)	9	3.7	0.009
2	canis,mus	380087	hg(352)	rn(982)	5	5.7	0.677
2	canis,rn	369584	hg(332)	mus(383)	2	1.6	0.453
3	hg,mus	406346	canis(599)	rn(858)	16	7.5	0.006
3	hg,rn	394036	canis(566)	mus(311)	9	2.4	0
3	canis,mus	378865	hg(303)	rn(797)	5	4.4	0.423
3	canis,rn	364549	hg(283)	mus(257)	2	1.1	0.31

For Rank differential = 2, the table shows the number of breaks in the variable species, the number of joint breaks, the average number of joint breaks in 1000 random shuffles and the p-value of the joint breaks, for FBP. This is based on greater than 500 bps markers common to Human, Mouse, Rat, and Dog. 4 of the 8 combinations have significant joint breaks.

### Analysis of correlated breaks in human

The regions that undergo repeated breaks in independent lineages are likely to be vulnerable to breakages. To further characterize these fragile regions, we compiled a set of 66 regions in the human genome which were not disrupted relative to mouse (respectively, rat) and was disrupted relative to rat (mouse) as well as relative to dog. In other words, each of these regions underwent independent breaks in both the rat (mouse) and dog lineage. Even though these regions were not disrupted in the human lineage (at least based on the marker set we have used), we investigated whether these 66 fragile regions (see Table S3 in Additional file 1) in human correlated with other indicators of fragility, *i.e*., segmental duplications and interspersed repeat sequences [9]. This examination of fragile regions in the genome in which they were not broken attempts to minimize the effect of negative selection as the potential reason for observed fragility. We extracted the segmental duplications for hg17 from the UCSC database. Also recall that the segmental duplications do not overlap the markers used to determine fragile regions.

**Figure 4:**
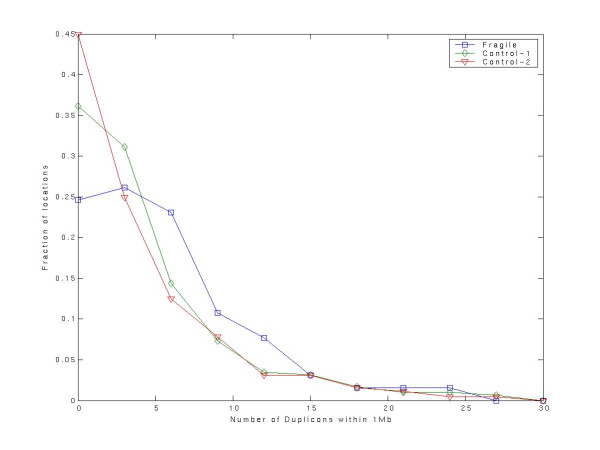
The number of segmental duplications within 1 Mb of potentially fragile regions in human relative to random locations in the genome (Control-1) and blocks in human genome that are syntenic in at least one another species and disrupted in at least one another species and excluding the fragile regions (Control-2). There are more segmental duplications near the fragile regions relative to Control-1 (Wilcoxon rank sum p-value = 0.0054) as well as relative to Control-2 (p-value = 0.00015).

We tested whether the number of segmental duplications within 1 Mb of a fragile region is greater than expected for a control. We selected two distinct controls. As Control-1, we randomly selected 6600 locations in the human genome. As a more stringent Control-2 we compiled 455 human regions which were broken in at least one other species and syntenic in at least one another species (the latter condition goes without saying because we only consider blocks are syntenic in at least 2 species), not including the 66 fragile regions. Figure 4 shows the distributions of number of segmental duplications in a 1 Mb vicinity for the 66 fragile regions, for the 6600 Control-1 locations, and for the 455 Control-2 regions. The fragile regions have a greater number of neighboring segmental duplications relative to Control-1 (*Wilcoxon rank sum *p-value = 0.0045), as well as relative to Control-2 (0.00015). The significance hold for a range of window sizes greater than 500 kb and less than 5 Mb. A similar test for the density of interspersed repeats showed significance only for Control-1 (*Wilcoxon rank sum *p-value = 0.0002) and not for Control-2.

## Discussion

Using a novel approach, we have shown that breakage events of a genomic region in independent lineages are significantly correlated, thus indicating that the predisposition of a genomic region for breakage is conserved over long evolutionary distances. Our results do not quantify the frequency of reuse, and do not necessarily imply that reuse is common. They do imply that however uncommon reuse may in fact be, it is still significantly more frequent than expected by chance.

### Robustness

Most of the early analyses of genomic rearrangements used very large markers (typically mega bases long) and only upon considering the finer resolution comparative genomic maps was it observed that the block size distribution did not follow the theoretical distribution based on uniform random breakage [7]. We thus feel compelled to base our analysis on the highest resolution data available to us. Although we have taken several precautions to minimize the possibility of wrong inferences regarding genomic breakages, we recognize that our analysis is limited by the quality of genome assembly and alignment technologies. Assembly errors, especially in genomes assembled using Whole Genome Shotgun, are widely known to occur, particularly near repeated and duplicated sequence. The problem is especially acute in case of the current chimpanzee build. If we attempt to ensure greater assembly accuracy by restricting ourselves to finished regions of each genome, however, we may not have enough data for statistical significance.

Multiple genome alignment can be seen as a consensus approach and relative to pair-wise genome alignment is likely to be more specific. By using various projections of the 8-way alignment, we hope to obtain a reliable alignment. The whole genome alignment used in this study was computed using Multiz alignment after repeat-masking the sequences to minimize misalignments. We further strengthen this by excluding markers that overlap low copy repeats or segmental duplication. Besides requiring a minimum marker length, we have employed three additional criteria. First, we require that the inter-marker distances have relatively similar lengths in the two species. Second, we require that a break be flanked by anchoring syntenic blocks with a minimum size threshold. And lastly, for reasons mentioned earlier, we also require that the inside-markers of the flanking blocks be sufficiently far from each other on the variable species to be considered a break. All of these stringency parameters, although reasonable, have the effect of reducing the number of observations, and below a critical number of observations it is hard to yield statistical significance. We note that over the space of considered parameters, as long as the number of observations falls above a critical limit, we observe significantly correlated breaks in a majority of the species combinations.

### Fragile regions versus negative selection

Many of the markers are likely to be functional elements, as evidenced by their conservation over long evolutionary periods. Furthermore, some of them might be constrained in a way that an evolutionary breakage disrupting their continuity or location will be selected against and therefore will not be in the population. A subset of the ones which do get fixed (and thus visible to us), perhaps by genetic drift, are minimally affected by the rearrangements and are thus likely to be non-functional or 'neutral', such as pseudogenes, repeats, segmental duplications of non-functional regions, and short genes that exist in high copy number. This is consistent with reports in the literature that associate evolutionary breakpoints with duplications, tRNA genes, repeated elements, etc. [19]. This raises the question of whether observed heterogeneity is evidence of a non-uniform process or rather of multiple constraints and purifying selection. Different responses to breakage in constrained and unconstrained regions may result in misleading conclusions regarding the prevalence of fragile regions. To test this possibility, as genes are obvious candidates for such constrained functional elements, we excluded blocks overlapping gene boundaries from consideration. Eliminating genic blocks should therefore have homogenized the results. In fact, however, inclusion of genic blocks in our analysis did not change our conclusion quantitatively (results not shown). Thus our conclusions seem to be independent of the functional nature of the markers.

### The root and the topology of evolutionary tree

The root of the evolutionary tree is an unresolved issue; however, our analysis is not sensitive to the placement of root since we are only concerned about the tree edges and paths between nodes, regardless of the direction. Thus we have intentionally presented the tree in Figure 1 as an unrooted tree. However, our method does depend on the topology of the evolutionary tree to infer independence of breakage events. Especially relevant to our work is the relationship between human, rodents and the dog. Rodents have evolved much faster than human and dogs and thus a sequence based tree reconstruction can sometimes reveal a ((Human, Dog), Rodent) relationship [20]. However, it is also believed that humans diverged from dogs earlier than the divergence of rodents and this was shown in two separate reports – based on common ancestral repeats [21] , and based on a selected set of 18 gene segments [22]. The same ((Human, Rodent), Dog) relationship is used in the public Dog genome paper [23]. Thus, we chose to use the ((Human, Rodent), Dog) relationship for our analysis.

## Conclusion

Fragility of a region is likely to be determined by several factors. Our findings, although not identifying these factors, do show that a particular region's probability of breakage is correlated with that of its homologous region in another organism. We do this by placing a breakage or disruption of a syntenic region unambiguously onto a lineage in the evolutionary tree.

An equally important question, not addressed in this work, is whether a particular region is more or less likely to break than any other region in the same organism. In our approach, although the density of joint breaks along the chromosome might intuitively be seen as such a measure, the observed number of joint breaks along any given chromosome is not high enough to reliably support such an interpretation. Others have attempted to address this by correlating the evolutionary breaks with other genomic landmarks as well as with breaks in cancer cells [5,24]. Even in the case of cancer genomes, however, the reoccurring breaks in independent samples do not rule out possible negative selection. Our observation that the fragile regions in the human genome are correlated with segmental duplications suggests that negative selection is unlikely to be the sole source of observed variations in fragility, assuming the segmental duplications found in the human genome are not conserved in those other species in which these regions are in fact disrupted.

## Methods

### Obtaining the syntenic markers

Unique, undisturbed stretches of sequence inherited from a common ancestor serve as genomic markers. Such markers are typically identified either by global multiple sequence alignment algorithms, or simply using reciprocal best matches using local alignment tools like BLAST. The multiple 8-way genomic alignment based on *Human(hg17), Chimpanzee(panTro1), Mouse(mm5), Rat(rn3), Dog(canFam1), and Chicken(galGal2), zebrafish(danRer1)*, and *fugufr1) *was obtained from the May 2004 release of UCSC genome resource [25]. This alignment is based on the *Multiz *alignment algorithm [17]. The chaining step of the algorithm results in ordered sequence of pair-wise nucleotide alignments or high scoring pairs (HSPs) separated by larger gaps [26]. The HSPs represent unique mapping across genomes and serve as the *syntenic markers *for our purposes. Any markers which overlapped another marker were removed, as were markers with non-unique mappings. We only considered markers on chromosomes 1 through 22 with precise location mapped. We took the projection of human, chimpanzee, mouse, rat, dog, and chicken from this 8-way alignment to obtain our Marker set. After taking the 6-way projection from the 8-way alignment we merge the juxtaposed blocks into single markers. We have also done the analysis based on a 5-way projection and 4-way projection as mentioned in the results. Finally all blocks whose location in the human genome overlapped with a segmental duplication were excluded. This was a precautionary measure to minimize the number of misaligned markers. Segmental duplication data was downloaded from the UCSC resource [27] and was based on methods described in [24]. Refseq annotations were also obtained from UCSC database. We observed that some markers were not unique across genomes (presumably paralogs), and we removed all markers that had such non-unique mappings.

### Identifying blocks and breaks

Given a set of *fixed *species and the *variable *species, we scan the markers ordered by genomic location on one of the fixed species to identify blocks of markers that are present in the same order and orientation in the other fixed species. The blocks can be of fixed size (4 or 6) for FBP or maximal syntenic blocks for FBR. For each such block, the order of markers in the variable species is examined to determine whether the block was broken or not (*i.e*., 1 or 0) based on the definition of a break, provided in results. This provides a binary table (Break-Table or *BT*) with *s *rows and 2 columns, where *s *is the number of identified blocks. The entry in *i^th ^*row and say 1^st ^column – *BT [i,1] *– is 1 if the *i^th ^*block is broken in the first variable species and 0 otherwise (likewise for 2^nd ^column).

### Significance of joint breaks using length-restricted permutation test

The *i^th ^*block has a *joint break *in the two variable species if *BT [i,1] *= *BT [i,2] *= 1. Recall that based on our parsimony assumption, breaks in the two variable species must have occurred independently. Hence Pr(*BT [i,1] *= 1 AND *BT [i,2] *= 1) = Pr(*BT [i,1] *= 1) * Pr(*BT [i,2] *= 1). There is no clear way to directly compute Pr(*BT [i,1] *= 1) since we do not know all the determinants of a break and the precise dependence of the break probability on those determinants. However in a model where the probability of breakage is uniform at every base pair, over evolutionary time, long segments are more likely to break than short segments. A straightforward analysis of breakpoint correlation would therefore reveal that breakage propensities are correlated in different lineages simply because longer segments will break more frequently in both lineages. We tested the "independent breakage" hypothesis by calculating a test statistic *β*, the count of joint breaks in homologous blocks, and computing its significance based on the values of *β *in a 1000 random matching of blocks across the two variable species in a length-restricted fashion. Each block has an associated breakpoint region length which is simply the distance between the flanks. Consider a syntenic block pair (x,y) in the two variable species. To randomize the pairs in a length-restricted fashion, we arbitrarily choose a species, say the first, and match block x with another randomly chosen block y' in the other species such that length(y') is within 20% or 200 bps of length(y) (whichever is greater). Thus in each randomization we replace each original block pair by another block pair of comparable length. We generate 1000 randomized sets of block-pairs. Thus the number of joint breaks is computed for each randomized set and the fraction of randomizations where the number of joint breaks exceeds *β *provides an estimate of the p-value of *β*, or *β^p^*.

## Authors' contributions

Both authors were involved in developing the idea and writing the manuscript. All analysis was done by HH.

## Supplementary Material

Additional File 1 Supplementary methods and results. The file contains the supplementary methods and results including the 3 supplementary tables. Click here for file
