# Supplementary material for: Recurring genomic breaks in independent lineages support genomic fragility
Source: BMC Evol Biol. 2006 Nov 7;6:90. doi: 10.1186/1471-2148-6-90 (PMC1636669; doi:10.1186/1471-2148-6-90)
Supplement: Additional File 1 — Supplementary methods and results. The file contains the supplementary methods and results including the 3 supplementary tables. [file 1471-2148-6-90-S1.doc]

## Supplementary methods and results

## Recurring genomic breaks in independent lineages support genomic fragility

## Hanno Hinsch and Sridhar Hannenhalli

***Estimating the significance of correlated breaks in independent lineages*- *length-restricted, directional shuffle***

**Method**

Here we present an alternative randomization strategy to assess the significance of joint breaks. Recall that each of the *s* blocks corresponds to a binary tuple - (b_1_ b_2_) – where b_1_ (respectively b_2_) is 1 if the block was broken in variable species 1 (respectively 2) and 0 otherwise. Given the vector of such binary tuples, we want to assess the significance of joint breaks *β, * i.e., the number of (1,1) tuples. The probability of a (1,1) tuple increases with the length of the breakpoint region. To account for this length dependence in our permutation test, we first sort the *s* tuples by the genomic length of the breakpoint regions of their corresponding blocks. We then generate a new set of tuples, creating each new tuple by pairing the first element of the original with an alternative block whose length is strictly greater than the original matching block (thereby making our test conservative). All blocks are sorted by their breakpoint region length and are assigned a rank. We create the first control set by matching the blocks from each tuple to the one whose length-based rank is 1 greater than the original. The next control set is created by matching to the block whose length-based rank is 2 greater than the original, and so on. As the number of samples increases, the blocks are compared to other blocks of ever larger length, making the test increasingly stringent. To strike a balance, we only compare blocks whose length rank is at most 200 greater than the original, which keeps the stringency in check while providing enough shuffles to establish statistical significance. Figure S1 illustrates this scheme. Finally, the number of times, among 200 shuffles, that the calculated number of joint breaks exceeds *β* provides a measure of significance *β^p^*. For instance if this number exceeds *β*  only once among 200 shuffle then *β^p^* = 0.005. We consider a correlated break frequency *significant* if *β^p^* ≤ 0.05. Next we describe the method in more detail.

The *i^th^* block has a *joint break* in the two variable species if *BT[i,1]* = *BT[i,2]* = 1. Recall that based on our parsimony assumption, breaks in the two variable species must have occurred independently. Hence Pr(*BT[i,1]*=1 AND *BT[i,2]*=1) = Pr(*BT[i,1]* =1) * Pr(*BT[i,2]*=1). There is no clear way to directly compute Pr(*BT[i,1]* =1) since we do not know all the determinants of a break and the precise dependence of the break probability on those determinants. However in a model where the probability of breakage is uniform at every base pair, over evolutionary time, long segments are more likely to break than short segments. A straightforward analysis of breakpoint correlation would therefore reveal that breakage propensities are correlated in different lineages simply because longer segments will break more frequently in both lineages. We tested the “independent breakage” hypothesis by calculating a test statistic *β*, the count of joint breaks in homologous blocks, and computing its significance based on the values of *β* in a series of length-restricted and directional shuffles, as described below. The fraction of times among 200 shuffles that the test statistic equals or exceeds *β* estimates the probability under the null hypothesis, and if this probability is less than 0.05, the assumption of independence can be rejected.

We sort the blocks in increasing order by their lengths in base pairs, *ie.*, length of block *i* ≤ length of block *i+k*, for *k* ≥ 0. From the monotonic relation between length of a region and its breakage probability, Pr(*BT[i,2]* =1) ≤ Pr(*BT[i+k,2]* =1). This leads to

Pr(*BT[i,1]* =1) * Pr(*BT[i,2]*=1) ≤ Pr(*BT[i,1]* =1) * Pr(*BT[i+k,2]*=1). In other words by ‘matching’ the *i^th^* block in the first variable species with the *(i+k)^th^* block in the second variable species the probability of joint break should *increase*. The *k^th^* ‘shuffle’ is defined as a matching of *i^th^* block in the first variable species to the *(i+k)^th^* block in the other variable species (Figure S1). We use 200 shuffles for 1 ≤ *k* ≤ 200. Note that this is not a randomized shuffle, but a deterministic sampling of matching in the length-neighborhood of blocks in a directional or asymmetric fashion. Another way to look at this is that in any given shuffle, each matching in the actual data is ‘replaced’ by another matching with a higher *a priori* probability of joint break. To avoid edge effects, the *k* longest blocks are disregarded. The distribution of joint breaks against which we compare our test statistic is composed entirely of pairs whose probability of a joint break, under the null hypothesis, is higher than that of the corresponding pairs used in calculating the observed test statistic. Thus the number of joint breaks is computed for each shuffle and the fractions of shuffles where the number of joint breaks exceeds *β* provides the lower bound on the p-value of *β*, or *β^p^*.


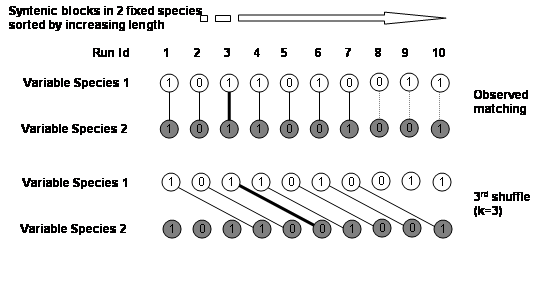


Figure S1. The shuffling scheme used to estimate the significance of joint break frequency. Here we use 10 blocks. For the 3^rd^ shuffle, the i^th^ block in the first variable species is matched with the (i+3)^rd^ block. The bold line indicates that the 3-3 pair in the actual data is ‘replaced’ with the 3-6 pair in the shuffle. The dashed lines show pairs excluded from the analysis to prevent edge effects. The number of observed joint breaks β, is 3, while the number of joint breaks in the shuffle is 2.

**Results using the directed shuffle**

*Correlated breaks using markers common to Human, Chimpanzee, Dog, Mouse, Rat, and Chicken.* Similar to the permutation test reported in the main text, we performed a total of 88 experiments with FBP and FBR (2 parameters and 44 species combinations). As before we require a minimum of 10 breaks in each of the variable species. In case of FBP, of the 63 combinations that meet this criterion, 40% have significantly correlated breaks (compared with 48% with the permutation test). In FBR, of the qualifying 46 combinations 17% are significant (compared with 30% with the permutation test).

*Correlated breaks using markers common to Human, Mouse, Rat, Dog, and Chicken.* There are a total of 40 experiments performed for each model (2 parameters and 20 species combinations). Using FBP, of the 33 qualifying combinations 39% are significant (compared with 47% with the permutation test), and using FBR, of the 20 qualifying combinations 25% are significant (compared with 33% with the permutation test). For a less restrictive *Minimum flank* = 2 for FBP, 50% of the 20 qualifying combinations are significant (compared with 55% with the permutation test).

*Correlated breaks using markers common to Human, Mouse, Rat, and Dog.* For *Rank Differential* threshold of 2, for FBP, 3 of the 8 combinations are highly significant (compared with 4 with the permutation test).

**Supplementary Tables**

***Table S1a.***

| **Minimum Flank** | **Fixed species** | **# runs** | **Variable species 1 (# breaks)** | **Variable species 2 (# breaks)** | **#joint breaks (*β*** | **Avg #joint breaks in shuffles** | **p-value of correlated break (*β^p^*)** |
| --- | --- | --- | --- | --- | --- | --- | --- |
| 2 | pan,mus | 108514 | rn(369) | hg(2) | 0 | 0.1 | 1 |
| 2 | pan,rn | 105221 | mus(74) | hg(3) | 0 | 0 | 1 |
| 2 | pan,gal | 50578 | canis(13) | mus(39) | 0 | 0.1 | 1 |
| 2 | pan,gal | 50578 | canis(13) | rn(97) | 1 | 0.2 | 0.18 |
| 2 | pan,gal | 50578 | canis(13) | hg(0) | 0 | 0 | 1 |
| 2 | pan,gal | 50578 | hg(0) | rn(97) | 0 | 0 | 1 |
| 2 | pan,gal | 50578 | hg(0) | mus(39) | 0 | 0 | 1 |
| 2 | pan,canis | 115359 | gal(1200) | mus(209) | 31 | 11.9 | 0 |
| 2 | pan,canis | 115359 | gal(1200) | rn(516) | 60 | 27.5 | 0 |
| 2 | pan,canis | 115359 | gal(1200) | hg(5) | 0 | 0.3 | 1 |
| 2 | pan,canis | 115359 | hg(5) | rn(516) | 0 | 0.1 | 1 |
| 2 | pan,canis | 115359 | hg(5) | mus(209) | 0 | 0 | 1 |
| 2 | hg,mus | 115287 | pan(281) | rn(399) | 7 | 2.5 | 0.013 |
| 2 | hg,mus | 115287 | pan(281) | gal(1144) | 14 | 6 | 0.004 |
| 2 | hg,mus | 115287 | pan(281) | canis(117) | 0 | 0.7 | 1 |
| 2 | hg,rn | 111697 | pan(272) | mus(72) | 0 | 0.4 | 1 |
| 2 | hg,rn | 111697 | pan(272) | gal(1084) | 12 | 5.6 | 0.006 |
| 2 | hg,rn | 111697 | pan(272) | canis(99) | 0 | 0.5 | 1 |
| 2 | hg,gal | 53668 | pan(104) | rn(109) | 1 | 0.6 | 0.453 |
| 2 | hg,gal | 53668 | pan(104) | mus(47) | 0 | 0.2 | 1 |
| 2 | hg,gal | 53668 | pan(104) | canis(14) | 0 | 0.1 | 1 |
| 2 | hg,canis | 122499 | pan(305) | rn(555) | 7 | 3.5 | 0.06 |
| 2 | hg,canis | 122499 | pan(305) | mus(223) | 0 | 1.3 | 1 |
| 2 | hg,canis | 122499 | pan(305) | gal(1238) | 15 | 6.7 | 0.006 |
| 3 | pan,mus | 105037 | rn(285) | hg(1) | 0 | 0 | 1 |
| 3 | pan,rn | 100936 | mus(58) | hg(1) | 0 | 0 | 1 |
| 3 | pan,gal | 42397 | canis(8) | mus(30) | 0 | 0.1 | 1 |
| 3 | pan,gal | 42397 | canis(8) | rn(71) | 1 | 0.2 | 0.149 |
| 3 | pan,gal | 42397 | canis(8) | hg(0) | 0 | 0 | 1 |
| 3 | pan,gal | 42397 | hg(0) | rn(71) | 0 | 0 | 1 |
| 3 | pan,gal | 42397 | hg(0) | mus(30) | 0 | 0 | 1 |
| 3 | pan,canis | 110094 | gal(766) | mus(160) | 14 | 7.5 | 0.023 |
| 3 | pan,canis | 110094 | gal(766) | rn(394) | 32 | 17.1 | 0 |
| 3 | pan,canis | 110094 | gal(766) | hg(3) | 0 | 0.2 | 1 |
| 3 | pan,canis | 110094 | hg(3) | rn(394) | 0 | 0.1 | 1 |
| 3 | pan,canis | 110094 | hg(3) | mus(160) | 0 | 0 | 1 |
| 3 | hg,mus | 112795 | pan(175) | rn(301) | 5 | 1.3 | 0.007 |
| 3 | hg,mus | 112795 | pan(175) | gal(742) | 4 | 2.8 | 0.309 |
| 3 | hg,mus | 112795 | pan(175) | canis(81) | 0 | 0.3 | 1 |
| 3 | hg,rn | 108197 | pan(174) | mus(62) | 0 | 0.3 | 1 |
| 3 | hg,rn | 108197 | pan(174) | gal(703) | 4 | 2.7 | 0.275 |
| 3 | hg,rn | 108197 | pan(174) | canis(71) | 0 | 0.3 | 1 |
| 3 | hg,gal | 45670 | pan(59) | rn(76) | 0 | 0.4 | 1 |
| 3 | hg,gal | 45670 | pan(59) | mus(33) | 0 | 0.2 | 1 |
| 3 | hg,gal | 45670 | pan(59) | canis(5) | 0 | 0 | 1 |
| 3 | hg,canis | 118105 | pan(191) | rn(417) | 5 | 1.8 | 0.034 |
| 3 | hg,canis | 118105 | pan(191) | mus(174) | 0 | 0.8 | 1 |
| 3 | hg,canis | 118105 | pan(191) | gal(791) | 4 | 3.3 | 0.412 |
| 2 | hg,mus | 115287 | canis(117) | rn(399) | 4 | 2.7 | 0.278 |
| 2 | hg,rn | 111697 | canis(99) | mus(72) | 1 | 0.4 | 0.322 |
| 2 | canis,mus | 111174 | hg(138) | rn(404) | 8 | 3.8 | 0.032 |
| 2 | canis,rn | 106992 | hg(132) | mus(69) | 4 | 0.5 | 0.001 |
| 2 | hg,mus | 115287 | gal(1144) | rn(399) | 32 | 18.7 | 0.001 |
| 2 | hg,rn | 111697 | gal(1084) | mus(72) | 7 | 2.8 | 0.022 |
| 2 | gal,mus | 42630 | hg(29) | rn(57) | 0 | 0.1 | 1 |
| 2 | gal,rn | 41642 | hg(31) | mus(17) | 1 | 0.1 | 0.071 |
| 2 | canis,mus | 111174 | gal(1192) | rn(404) | 34 | 20.6 | 0.004 |
| 2 | canis,rn | 106992 | gal(1131) | mus(69) | 7 | 2.9 | 0.027 |
| 2 | gal,mus | 42630 | canis(19) | rn(57) | 1 | 0.2 | 0.172 |
| 2 | gal,rn | 41642 | canis(18) | mus(17) | 0 | 0 | 1 |
| 2 | canis,hg | 112688 | gal(1216) | rn(548) | 67 | 29.7 | 0 |
| 2 | canis,rn | 106992 | gal(1131) | hg(132) | 15 | 7.6 | 0.01 |
| 2 | gal,hg | 34686 | canis(14) | rn(53) | 0 | 0 | 1 |
| 2 | gal,rn | 41642 | canis(18) | hg(31) | 3 | 0 | 0 |
| 2 | canis,hg | 112688 | gal(1216) | mus(219) | 39 | 12.2 | 0 |
| 2 | canis,mus | 111174 | gal(1192) | hg(138) | 19 | 8.4 | 0.001 |
| 2 | gal,hg | 34686 | canis(14) | mus(25) | 0 | 0 | 1 |
| 2 | gal,mus | 42630 | canis(19) | hg(29) | 3 | 0 | 0 |
| 3 | hg,mus | 112795 | canis(81) | rn(301) | 3 | 1.8 | 0.282 |
| 3 | hg,rn | 108197 | canis(71) | mus(62) | 1 | 0.4 | 0.311 |
| 3 | canis,mus | 109430 | hg(103) | rn(311) | 7 | 2.5 | 0.008 |
| 3 | canis,rn | 104234 | hg(96) | mus(59) | 2 | 0.5 | 0.082 |
| 3 | hg,mus | 112795 | gal(742) | rn(301) | 20 | 11.4 | 0.012 |
| 3 | hg,rn | 108197 | gal(703) | mus(62) | 6 | 2 | 0.016 |
| 3 | gal,mus | 34491 | hg(15) | rn(33) | 0 | 0.1 | 1 |
| 3 | gal,rn | 33201 | hg(17) | mus(12) | 1 | 0 | 0.026 |
| 3 | canis,mus | 109430 | gal(792) | rn(311) | 22 | 13 | 0.007 |
| 3 | canis,rn | 104234 | gal(741) | mus(59) | 4 | 2.2 | 0.201 |
| 3 | gal,mus | 34491 | canis(9) | rn(33) | 1 | 0.1 | 0.09 |
| 3 | gal,rn | 33201 | canis(8) | mus(12) | 0 | 0.1 | 1 |
| 3 | canis,hg | 110296 | gal(803) | rn(421) | 38 | 19 | 0.001 |
| 3 | canis,rn | 104234 | gal(741) | hg(96) | 9 | 5 | 0.075 |
| 3 | gal,hg | 25487 | canis(8) | rn(29) | 0 | 0 | 1 |
| 3 | gal,rn | 33201 | canis(8) | hg(17) | 0 | 0 | 1 |
| 3 | canis,hg | 110296 | gal(803) | mus(177) | 20 | 8.7 | 0 |
| 3 | canis,mus | 109430 | gal(792) | hg(103) | 11 | 5.3 | 0.015 |
| 3 | gal,hg | 25487 | canis(8) | mus(11) | 0 | 0 | 1 |
| 3 | gal,mus | 34491 | canis(9) | hg(15) | 0 | 0 | 1 |

*Based on common markers in Human, Chimp, Mouse, rat, Dog, and Chicken, for the 2 values of Minimum flank (Rank differential = 3) the table shows the number of breaks in the variable species, the number of joint breaks, the average number of joint breaks in 1000 random shuffles and the p-value of the joint breaks for FBP. There are 63 combinations in which both variable species have at least 10 breaks. Among these 63 cases 30 (48%) exhibit significant joint breaks.*

***Table S1b.***

| **Minimum Flank** | **Fixed species** | **# runs** | **Variable species 1 (# breaks)** | **Variable species 2 (# breaks)** | **#joint breaks (*β*** | **Avg #joint breaks in shuffles** | **p-value of correlated break (*β^p^*)** |
| --- | --- | --- | --- | --- | --- | --- | --- |
| 2 | pan,mus | 12666 | rn(166) | hg(1) | 0 | 0.1 | 1 |
| 2 | pan,rn | 12675 | mus(35) | hg(1) | 0 | 0 | 1 |
| 2 | pan,gal | 3227 | canis(1) | mus(0) | 0 | 0 | 1 |
| 2 | pan,gal | 3227 | canis(1) | rn(4) | 0 | 0 | 1 |
| 2 | pan,gal | 3227 | canis(1) | hg(0) | 0 | 0 | 1 |
| 2 | pan,gal | 3227 | hg(0) | rn(4) | 0 | 0 | 1 |
| 2 | pan,gal | 3227 | hg(0) | mus(0) | 0 | 0 | 1 |
| 2 | pan,canis | 12476 | gal(637) | mus(115) | 18 | 14 | 0.158 |
| 2 | pan,canis | 12476 | gal(637) | rn(266) | 50 | 32.6 | 0.001 |
| 2 | pan,canis | 12476 | gal(637) | hg(3) | 1 | 0.3 | 0.261 |
| 2 | pan,canis | 12476 | hg(3) | rn(266) | 0 | 0.1 | 1 |
| 2 | pan,canis | 12476 | hg(3) | mus(115) | 0 | 0 | 1 |
| 2 | hg,mus | 12616 | pan(80) | rn(187) | 4 | 2.4 | 0.22 |
| 2 | hg,mus | 12616 | pan(80) | gal(505) | 8 | 6 | 0.238 |
| 2 | hg,mus | 12616 | pan(80) | canis(52) | 0 | 0.7 | 1 |
| 2 | hg,rn | 12673 | pan(94) | mus(35) | 0 | 0.4 | 1 |
| 2 | hg,rn | 12673 | pan(94) | gal(476) | 9 | 6 | 0.134 |
| 2 | hg,rn | 12673 | pan(94) | canis(44) | 0 | 0.6 | 1 |
| 2 | hg,gal | 3414 | pan(1) | rn(5) | 0 | 0 | 1 |
| 2 | hg,gal | 3414 | pan(1) | mus(1) | 0 | 0 | 1 |
| 2 | hg,gal | 3414 | pan(1) | canis(1) | 0 | 0 | 1 |
| 2 | hg,canis | 12081 | pan(119) | rn(299) | 12 | 6.1 | 0.013 |
| 2 | hg,canis | 12081 | pan(119) | mus(132) | 6 | 2.6 | 0.04 |
| 2 | hg,canis | 12081 | pan(119) | gal(692) | 22 | 11.9 | 0.002 |
| 3 | pan,mus | 6731 | rn(69) | hg(1) | 0 | 0 | 1 |
| 3 | pan,rn | 6603 | mus(15) | hg(1) | 0 | 0 | 1 |
| 3 | pan,gal | 546 | canis(0) | mus(0) | 0 | 0 | 1 |
| 3 | pan,gal | 546 | canis(0) | rn(0) | 0 | 0 | 1 |
| 3 | pan,gal | 546 | canis(0) | hg(0) | 0 | 0 | 1 |
| 3 | pan,gal | 546 | hg(0) | rn(0) | 0 | 0 | 1 |
| 3 | pan,gal | 546 | hg(0) | mus(0) | 0 | 0 | 1 |
| 3 | pan,canis | 7942 | gal(266) | mus(46) | 7 | 3.4 | 0.049 |
| 3 | pan,canis | 7942 | gal(266) | rn(128) | 21 | 9.8 | 0 |
| 3 | pan,canis | 7942 | gal(266) | hg(1) | 0 | 0 | 1 |
| 3 | pan,canis | 7942 | hg(1) | rn(128) | 0 | 0 | 1 |
| 3 | pan,canis | 7942 | hg(1) | mus(46) | 0 | 0 | 1 |
| 3 | hg,mus | 6908 | pan(40) | rn(81) | 2 | 0.9 | 0.21 |
| 3 | hg,mus | 6908 | pan(40) | gal(184) | 3 | 1.7 | 0.242 |
| 3 | hg,mus | 6908 | pan(40) | canis(24) | 0 | 0.2 | 1 |
| 3 | hg,rn | 6853 | pan(38) | mus(15) | 0 | 0.1 | 1 |
| 3 | hg,rn | 6853 | pan(38) | gal(164) | 2 | 1.6 | 0.474 |
| 3 | hg,rn | 6853 | pan(38) | canis(17) | 0 | 0.2 | 1 |
| 3 | hg,gal | 588 | pan(0) | rn(1) | 0 | 0 | 1 |
| 3 | hg,gal | 588 | pan(0) | mus(0) | 0 | 0 | 1 |
| 3 | hg,gal | 588 | pan(0) | canis(0) | 0 | 0 | 1 |
| 3 | hg,canis | 7981 | pan(65) | rn(153) | 5 | 2.5 | 0.113 |
| 3 | hg,canis | 7981 | pan(65) | mus(60) | 3 | 1.2 | 0.133 |
| 3 | hg,canis | 7981 | pan(65) | gal(313) | 8 | 4.6 | 0.088 |
| 2 | hg,mus | 12616 | canis(52) | rn(187) | 3 | 3.1 | 0.608 |
| 2 | hg,rn | 12673 | canis(44) | mus(35) | 0 | 0.3 | 1 |
| 2 | canis,mus | 12817 | hg(41) | rn(198) | 3 | 2.3 | 0.404 |
| 2 | canis,rn | 12941 | hg(45) | mus(36) | 3 | 0.3 | 0.005 |
| 2 | hg,mus | 12616 | gal(505) | rn(187) | 37 | 19.8 | 0 |
| 2 | hg,rn | 12673 | gal(476) | mus(35) | 3 | 3.4 | 0.669 |
| 2 | gal,mus | 4659 | hg(6) | rn(13) | 0 | 0 | 1 |
| 2 | gal,rn | 4411 | hg(1) | mus(2) | 0 | 0 | 1 |
| 2 | canis,mus | 12817 | gal(597) | rn(198) | 36 | 23.3 | 0.006 |
| 2 | canis,rn | 12941 | gal(543) | mus(36) | 4 | 3.7 | 0.504 |
| 2 | gal,mus | 4659 | canis(5) | rn(13) | 1 | 0.1 | 0.131 |
| 2 | gal,rn | 4411 | canis(7) | mus(2) | 0 | 0 | 1 |
| 2 | canis,hg | 10629 | gal(875) | rn(379) | 94 | 72.4 | 0.003 |
| 2 | canis,rn | 12941 | gal(543) | hg(45) | 4 | 5.5 | 0.807 |
| 2 | gal,hg | 4894 | canis(5) | rn(12) | 0 | 0 | 1 |
| 2 | gal,rn | 4411 | canis(7) | hg(1) | 0 | 0 | 1 |
| 2 | canis,hg | 10629 | gal(875) | mus(189) | 57 | 36.3 | 0.001 |
| 2 | canis,mus | 12817 | gal(597) | hg(41) | 7 | 5.6 | 0.341 |
| 2 | gal,hg | 4894 | canis(5) | mus(1) | 0 | 0 | 1 |
| 2 | gal,mus | 4659 | canis(5) | hg(6) | 0 | 0 | 1 |
| 3 | hg,mus | 6908 | canis(24) | rn(81) | 1 | 0.9 | 0.595 |
| 3 | hg,rn | 6853 | canis(17) | mus(15) | 0 | 0.1 | 1 |
| 3 | canis,mus | 7379 | hg(27) | rn(94) | 1 | 1.3 | 0.751 |
| 3 | canis,rn | 7370 | hg(18) | mus(17) | 1 | 0.1 | 0.085 |
| 3 | hg,mus | 6908 | gal(184) | rn(81) | 10 | 5.1 | 0.03 |
| 3 | hg,rn | 6853 | gal(164) | mus(15) | 0 | 0.8 | 1 |
| 3 | gal,mus | 955 | hg(1) | rn(1) | 0 | 0 | 1 |
| 3 | gal,rn | 856 | hg(0) | mus(0) | 0 | 0 | 1 |
| 3 | canis,mus | 7379 | gal(235) | rn(94) | 11 | 6.5 | 0.07 |
| 3 | canis,rn | 7370 | gal(217) | mus(17) | 1 | 1 | 0.646 |
| 3 | gal,mus | 955 | canis(0) | rn(1) | 0 | 0 | 1 |
| 3 | gal,rn | 856 | canis(0) | mus(0) | 0 | 0 | 1 |
| 3 | canis,hg | 7919 | gal(460) | rn(222) | 44 | 27.4 | 0 |
| 3 | canis,rn | 7370 | gal(217) | hg(18) | 2 | 1.4 | 0.398 |
| 3 | gal,hg | 1049 | canis(0) | rn(1) | 0 | 0 | 1 |
| 3 | gal,rn | 856 | canis(0) | hg(0) | 0 | 0 | 1 |
| 3 | canis,hg | 7919 | gal(460) | mus(102) | 24 | 13 | 0.003 |
| 3 | canis,mus | 7379 | gal(235) | hg(27) | 4 | 2.7 | 0.262 |
| 3 | gal,hg | 1049 | canis(0) | mus(0) | 0 | 0 | 1 |
| 3 | gal,mus | 955 | canis(0) | hg(1) | 0 | 0 | 1 |

*Based on common markers in Human, Chimp, Mouse, rat, Dog, and Chicken, for the 2 values of Minimum flank (Rank differential = 3) the table shows the number of breaks in the variable species, the number of joint breaks, the number of joint breaks in 1000 random shuffles and the p-value of the joint breaks for FBR. There are 46 combinations in which both variable species have at least 10 breaks. Among these 46 cases 14 (30%) exhibit significant joint breaks.*

***Table S2a.***

| **Minimum Flank** | **Fixed species** | **# runs** | **Variable species 1 (# breaks)** | **Variable species 2 (# breaks)** | **#joint breaks (*β*** | **Avg #joint breaks in shuffles** | **p-value of correlated break (*β^p^*)** |
| --- | --- | --- | --- | --- | --- | --- | --- |
| 2 | hg,mus | 126798 | canis(127) | rn(421) | 3 | 2.4 | 0.441 |
| 2 | hg,rn | 122981 | canis(111) | mus(69) | 0 | 0.3 | 1 |
| 2 | canis,mus | 121649 | hg(81) | rn(416) | 0 | 1.8 | 1 |
| 2 | canis,rn | 117204 | hg(84) | mus(69) | 1 | 0.3 | 0.286 |
| 2 | hg,mus | 126798 | gal(1224) | rn(421) | 33 | 19.1 | 0.002 |
| 2 | hg,rn | 122981 | gal(1152) | mus(69) | 6 | 2.6 | 0.036 |
| 2 | gal,mus | 47121 | hg(17) | rn(60) | 0 | 0.1 | 1 |
| 2 | gal,rn | 46049 | hg(19) | mus(19) | 1 | 0 | 0.01 |
| 2 | canis,mus | 121649 | gal(1253) | rn(416) | 35 | 19.8 | 0.001 |
| 2 | canis,rn | 117204 | gal(1187) | mus(69) | 8 | 3 | 0.011 |
| 2 | gal,mus | 47121 | canis(20) | rn(60) | 1 | 0.1 | 0.126 |
| 2 | gal,rn | 46049 | canis(19) | mus(19) | 1 | 0 | 0.047 |
| 2 | canis,hg | 123305 | gal(1278) | rn(582) | 67 | 30.5 | 0 |
| 2 | canis,rn | 117204 | gal(1187) | hg(84) | 9 | 4.4 | 0.05 |
| 2 | gal,hg | 38367 | canis(17) | rn(55) | 0 | 0 | 1 |
| 2 | gal,rn | 46049 | canis(19) | hg(19) | 3 | 0 | 0 |
| 2 | canis,hg | 123305 | gal(1278) | mus(229) | 37 | 12.3 | 0 |
| 2 | canis,mus | 121649 | gal(1253) | hg(81) | 8 | 4.4 | 0.081 |
| 2 | gal,hg | 38367 | canis(17) | mus(30) | 0 | 0.1 | 1 |
| 2 | gal,mus | 47121 | canis(20) | hg(17) | 1 | 0 | 0.021 |
| 3 | hg,mus | 124349 | canis(86) | rn(319) | 2 | 1.5 | 0.454 |
| 3 | hg,rn | 119466 | canis(76) | mus(61) | 0 | 0.3 | 1 |
| 3 | canis,mus | 120074 | hg(56) | rn(322) | 0 | 1.4 | 1 |
| 3 | canis,rn | 114517 | hg(53) | mus(62) | 1 | 0.3 | 0.244 |
| 3 | hg,mus | 124349 | gal(809) | rn(319) | 21 | 11.9 | 0.012 |
| 3 | hg,rn | 119466 | gal(764) | mus(61) | 5 | 1.9 | 0.033 |
| 3 | gal,mus | 38428 | hg(6) | rn(35) | 0 | 0 | 1 |
| 3 | gal,rn | 37048 | hg(7) | mus(12) | 0 | 0 | 1 |
| 3 | canis,mus | 120074 | gal(844) | rn(322) | 24 | 13 | 0.005 |
| 3 | canis,rn | 114517 | gal(792) | mus(62) | 5 | 2.3 | 0.078 |
| 3 | gal,mus | 38428 | canis(15) | rn(35) | 1 | 0.1 | 0.096 |
| 3 | gal,rn | 37048 | canis(10) | mus(12) | 0 | 0 | 1 |
| 3 | canis,hg | 121005 | gal(857) | rn(448) | 36 | 19 | 0.001 |
| 3 | canis,rn | 114517 | gal(792) | hg(53) | 4 | 2.6 | 0.279 |
| 3 | gal,hg | 28547 | canis(9) | rn(31) | 0 | 0 | 1 |
| 3 | gal,rn | 37048 | canis(10) | hg(7) | 0 | 0 | 1 |
| 3 | canis,hg | 121005 | gal(857) | mus(188) | 20 | 8.5 | 0 |
| 3 | canis,mus | 120074 | gal(844) | hg(56) | 4 | 2.9 | 0.336 |
| 3 | gal,hg | 28547 | canis(9) | mus(13) | 0 | 0.1 | 1 |
| 3 | gal,mus | 38428 | canis(15) | hg(6) | 0 | 0 | 1 |

*Based on common markers in Human, Mouse, rat, Dog, and Chicken, for the 2 values of Minimum flank (Rank differential = 3) the table shows the number of breaks in the variable species, the number of joint breaks, the number of joint breaks in 1000 random shuffles and the p-value of the joint breaks for FBP. There are 34 combinations in which both variable species have at least 10 breaks. Among these 34 cases 16 (47%) exhibit significant joint breaks.*

***Table S2b.***

| **Minimum Flank** | **Fixed species** | **# runs** | **Variable species 1 (# breaks)** | **Variable species 2 (# breaks)** | **#joint breaks (*β*** | **Avg #joint breaks in shuffles** | **p-value of correlated break (*β^p^*)** |
| --- | --- | --- | --- | --- | --- | --- | --- |
| 2 | hg,mus | 13822 | canis(53) | rn(197) | 3 | 3.2 | 0.633 |
| 2 | hg,rn | 13920 | canis(47) | mus(34) | 0 | 0.4 | 1 |
| 2 | canis,mus | 14104 | hg(23) | rn(209) | 3 | 1.7 | 0.226 |
| 2 | canis,rn | 14205 | hg(22) | mus(31) | 2 | 0.5 | 0.063 |
| 2 | hg,mus | 13822 | gal(538) | rn(197) | 41 | 21.5 | 0 |
| 2 | hg,rn | 13920 | gal(495) | mus(34) | 5 | 3.1 | 0.198 |
| 2 | gal,mus | 4898 | hg(3) | rn(11) | 0 | 0 | 1 |
| 2 | gal,rn | 4633 | hg(2) | mus(1) | 0 | 0 | 1 |
| 2 | canis,mus | 14104 | gal(635) | rn(209) | 37 | 23 | 0.004 |
| 2 | canis,rn | 14205 | gal(592) | mus(31) | 4 | 2.9 | 0.334 |
| 2 | gal,mus | 4898 | canis(4) | rn(11) | 0 | 0.1 | 1 |
| 2 | gal,rn | 4633 | canis(6) | mus(1) | 0 | 0 | 1 |
| 2 | canis,hg | 11895 | gal(901) | rn(416) | 90 | 75.4 | 0.048 |
| 2 | canis,rn | 14205 | gal(592) | hg(22) | 3 | 2.5 | 0.488 |
| 2 | gal,hg | 5196 | canis(4) | rn(13) | 0 | 0.1 | 1 |
| 2 | gal,rn | 4633 | canis(6) | hg(2) | 0 | 0 | 1 |
| 2 | canis,hg | 11895 | gal(901) | mus(198) | 47 | 35.8 | 0.032 |
| 2 | canis,mus | 14104 | gal(635) | hg(23) | 5 | 3 | 0.193 |
| 2 | gal,hg | 5196 | canis(4) | mus(3) | 0 | 0 | 1 |
| 2 | gal,mus | 4898 | canis(4) | hg(3) | 0 | 0 | 1 |
| 3 | hg,mus | 7535 | canis(23) | rn(86) | 1 | 1 | 0.648 |
| 3 | hg,rn | 7390 | canis(19) | mus(13) | 0 | 0.1 | 1 |
| 3 | canis,mus | 8076 | hg(14) | rn(86) | 2 | 0.3 | 0.041 |
| 3 | canis,rn | 7935 | hg(10) | mus(18) | 0 | 0 | 1 |
| 3 | hg,mus | 7535 | gal(205) | rn(86) | 12 | 5.6 | 0.011 |
| 3 | hg,rn | 7390 | gal(175) | mus(13) | 0 | 0.7 | 1 |
| 3 | gal,mus | 953 | hg(0) | rn(2) | 0 | 0 | 1 |
| 3 | gal,rn | 850 | hg(1) | mus(0) | 0 | 0 | 1 |
| 3 | canis,mus | 8076 | gal(232) | rn(86) | 9 | 5 | 0.058 |
| 3 | canis,rn | 7935 | gal(221) | mus(18) | 1 | 1.1 | 0.656 |
| 3 | gal,mus | 953 | canis(0) | rn(2) | 0 | 0 | 1 |
| 3 | gal,rn | 850 | canis(0) | mus(0) | 0 | 0 | 1 |
| 3 | canis,hg | 8724 | gal(454) | rn(243) | 42 | 27.7 | 0.002 |
| 3 | canis,rn | 7935 | gal(221) | hg(10) | 2 | 0.7 | 0.156 |
| 3 | gal,hg | 1078 | canis(0) | rn(1) | 0 | 0 | 1 |
| 3 | gal,rn | 850 | canis(0) | hg(1) | 0 | 0 | 1 |
| 3 | canis,hg | 8724 | gal(454) | mus(103) | 19 | 12.4 | 0.038 |
| 3 | canis,mus | 8076 | gal(232) | hg(14) | 2 | 1.1 | 0.292 |
| 3 | gal,hg | 1078 | canis(0) | mus(0) | 0 | 0 | 1 |
| 3 | gal,mus | 953 | canis(0) | hg(0) | 0 | 0 | 1 |

*Based on common markers in Human, Mouse, rat, Dog, and Chicken, for the 2 values of Minimum flank (Rank differential = 3) the table shows the number of breaks in the variable species, the number of joint breaks, the number of joint breaks in 1000 random shuffles and the p-value of the joint breaks for FBR. There are 24 combinations in which both variable species have at least 10 breaks. Among these 24 cases8 (33%) exhibit significant joint breaks.*

***Table S3.***

| **Chromosome** | **Begin** | **End** |
| --- | --- | --- |
| chr1 | 18419301 | 18454644 |
| chr1 | 31433803 | 31506081 |
| chr1 | 31497801 | 31509686 |
| chr1 | 40784164 | 40881048 |
| chr1 | 40902933 | 40908208 |
| chr1 | 43474550 | 43497032 |
| chr1 | 46797739 | 46813683 |
| chr1 | 47566535 | 47673275 |
| chr1 | 47614826 | 47673275 |
| chr1 | 87286534 | 87404806 |
| chr1 | 95069625 | 95077421 |
| chr1 | 155835587 | 155845980 |
| chr1 | 159298363 | 159314916 |
| chr1 | 163031582 | 163037758 |
| chr1 | 164667431 | 164703476 |
| chr1 | 178499058 | 179154843 |
| chr1 | 217293721 | 217358692 |
| chr1 | 217444478 | 217456246 |
| chr2 | 52623609 | 52723952 |
| chr2 | 69512576 | 69558417 |
| chr2 | 162153668 | 162173219 |
| chr2 | 207803712 | 207824359 |
| chr2 | 224425913 | 224449234 |
| chr3 | 180156612 | 180216514 |
| chr4 | 17284532 | 17309930 |
| chr4 | 57183750 | 57195112 |
| chr5 | 119109535 | 119121811 |
| chr5 | 133554190 | 133564720 |
| chr6 | 24524521 | 24539417 |
| chr6 | 34671438 | 34730593 |
| chr6 | 49475017 | 49486546 |
| chr6 | 145209539 | 145241540 |
| chr6 | 167156307 | 167388396 |
| chr7 | 4648982 | 4691015 |
| chr7 | 37553224 | 37608856 |
| chr7 | 68856566 | 69040601 |
| chr7 | 79491772 | 79935214 |
| chr7 | 79491772 | 79947251 |
| chr7 | 91670052 | 91722847 |
| chr7 | 133600715 | 133821096 |
| chr7 | 147550133 | 147888555 |
| chr7 | 154537576 | 154754856 |
| chr8 | 434485 | 604687 |
| chr8 | 33513689 | 33528810 |
| chr8 | 109770768 | 109852092 |
| chr9 | 133275047 | 133533007 |
| chr9 | 133303245 | 133319218 |
| chr10 | 70372918 | 70396986 |
| chr10 | 70652124 | 70999789 |
| chr10 | 76527444 | 76581014 |
| chr10 | 100603868 | 100626368 |
| chr11 | 5620931 | 5643632 |
| chr11 | 39296712 | 39324311 |
| chr12 | 127418173 | 127579614 |
| chr13 | 52680032 | 52739044 |
| chr13 | 63786494 | 63899092 |
| chr14 | 38785858 | 38852244 |
| chr17 | 3766813 | 3854657 |
| chr17 | 3766988 | 3854657 |
| chr17 | 10177105 | 10387214 |
| chr17 | 59912304 | 59936926 |
| chr18 | 47608284 | 47620247 |
| chr19 | 8575891 | 8648574 |
| chr19 | 13905514 | 14127487 |
| chr20 | 22751755 | 22790112 |
| chr20 | 23541487 | 23566060 |

*Regions in human genome (hg17) that were broken in dog as well as in exactly one of the rodents.*
